# Modeling changes in baleen whale seasonal abundance, timing of migration, and environmental variables to explain the sudden rise in entanglements in California

**DOI:** 10.1371/journal.pone.0248557

**Published:** 2021-04-15

**Authors:** Kaytlin Ingman, Ellen Hines, Piero L. F. Mazzini, R. Cotton Rockwood, Nadav Nur, Jaime Jahncke

**Affiliations:** 1 Point Blue Conservation Science, Petaluma, CA, United States of America; 2 Estuary & Ocean Science Center, SFSU, Tiburon, CA, United States of America; 3 Department of Geography & Environment, SFSU, San Francisco, CA, United States of America; 4 Virginia Institute of Marine Science, William & Mary, Gloucester Point, VA, United States of America; Institute of Deep-sea Science and Engineering, Chinese Academy of Sciences, CHINA

## Abstract

We document changes in the number of sightings and timing of humpback (*Megaptera novaeangliae*), blue (*Balaenoptera musculus*), and gray (*Eschrichtius robustus*) whale migratory phases in the vicinity of the Farallon Islands, California. We hypothesized that changes in the timing of migration off central California were driven by local oceanography, regional upwelling, and basin-scale climate conditions. Using 24 years of daily whale counts collected from Southeast Farallon Island, we developed negative binomial regression models to evaluate trends in local whale sightings over time. We then used linear models to assess trends in the timing of migration, and to identify potential environmental drivers. These drivers included local, regional and basin-scale patterns; the latter included the El Niño Southern Oscillation, the Pacific Decadal Oscillation, and the North Pacific Gyre Oscillation, which influence, wind-driven upwelling, and overall productivity in the California Current System. We then created a forecast model to predict the timing of migration. Humpback whale sightings significantly increased over the study period, but blue and gray whale counts did not, though there was variability across the time series. Date of breeding migration (departure) for all species showed little to no change, whereas date of migration towards feeding areas (arrival) occurred earlier for humpback and blue whales. Timing was significantly influenced by a mix of local oceanography, regional, and basin-scale climate variables. Earlier arrival time without concomitant earlier departure time results in longer periods when blue and humpback whales are at risk of entanglement in the Gulf of the Farallones. We maintain that these changes have increased whale exposure to pot and trap fishery gear off the central California coast during the spring, elevating the risk of entanglements. Humpback entanglement rates were significantly associated with increased counts and early arrival in central California. Actions to decrease the temporal overlap between whales and pot/trap fishing gear, particularly when whales arrive earlier in warm water years, would likely decrease the risk of entanglements.

## Introduction

The California Current System (CCS) is one of four highly productive wind-driven upwelling systems [1, 2] and an important destination for migrating marine megafauna [3–5]. Wind-driven upwelling brings cold, saline, nutrient-rich water to the surface in the spring and summer, enhancing both primary and secondary production, as well as attracting foraging top predators [1, 2, 4]. Variability in ocean conditions in the northeastern Pacific Ocean is mainly driven by the El Niño Southern Oscillation (ENSO), the Pacific Decadal Oscillation (PDO), and the North Pacific Gyre Oscillation (NPGO) [6–8]. These basin-scale climate patterns alter atmospheric circulation, wind patterns, and overall coastal upwelling strength, which in turn affects sea surface temperature (SST), sea surface salinity (SSS), nutrients, and productivity on annual to decadal timescales [7, 9–11]. Mid-trophic level species are highly susceptible to changes in water mass properties and productivity [5, 12]. Copepod (Family: Calanoidea) abundance and species composition in central California change in response to climate [13]. Krill (Family: Euphausiidae) abundance can decrease up to 30% in response to reduced upwelling and productivity [14]. Forage fish species, such as juvenile rockfish (Family: Scorpaenidae) and northern anchovy (*Engraulis mordax)*, also decline in abundance or shift distributions during poor upwelling years [12]. Changes in the prey field generally have negative consequences for higher trophic levels, including baleen whales [15].

Three baleen whale species migrate through central California: humpback (*Megaptera novaeangliae*), blue (*Balaenoptera musculus*), and gray (*Eschrichtius robustus)* whales. Blue whales come to the area to feed on krill and humpbacks come to feed on both krill and forage fish during the upwelling season in the summer and fall [15, 16]. These two species winter in their breeding grounds in the tropics [15, 16]. Though gray whales also winter in their breeding grounds at lower latitudes, central California is not a primary feeding ground [17]. They are commonly spotted on either their northward non-breeding migration for a few months in early spring, or on their southward breeding migration beginning in the fall [17].

Range shifts and expansions in cetacean species in the CCS, including the three species mentioned previously, have been documented [18–20]. For example, gray whales altered the timing of migration to decrease thermal stress during warm, unproductive periods, such as El Niño years [21, 22]. Blue whales changed migration routes, tracking their prey as krill abundance patterns changed with PDO phases [23]. Humpback whales modified the timing of their migration [21], followed prey patches [3], and switched prey when krill was less available in response to El Niño, warm-phase PDO, and the unproductive NPGO phase [24]. Identifying and understanding spatial and temporal patterns of behavior in these species contributes to the prediction and mitigation of emerging threats [25].

Baleen whales were commercially harvested and nearly globally eradicated by the early- to mid-20^th^ century [26]. Whales have been protected from harvest in the USA since 1972, and internationally by the International Whaling Commission since 1986, initiating the slow recovery of these populations [26].

**Fig 1:**
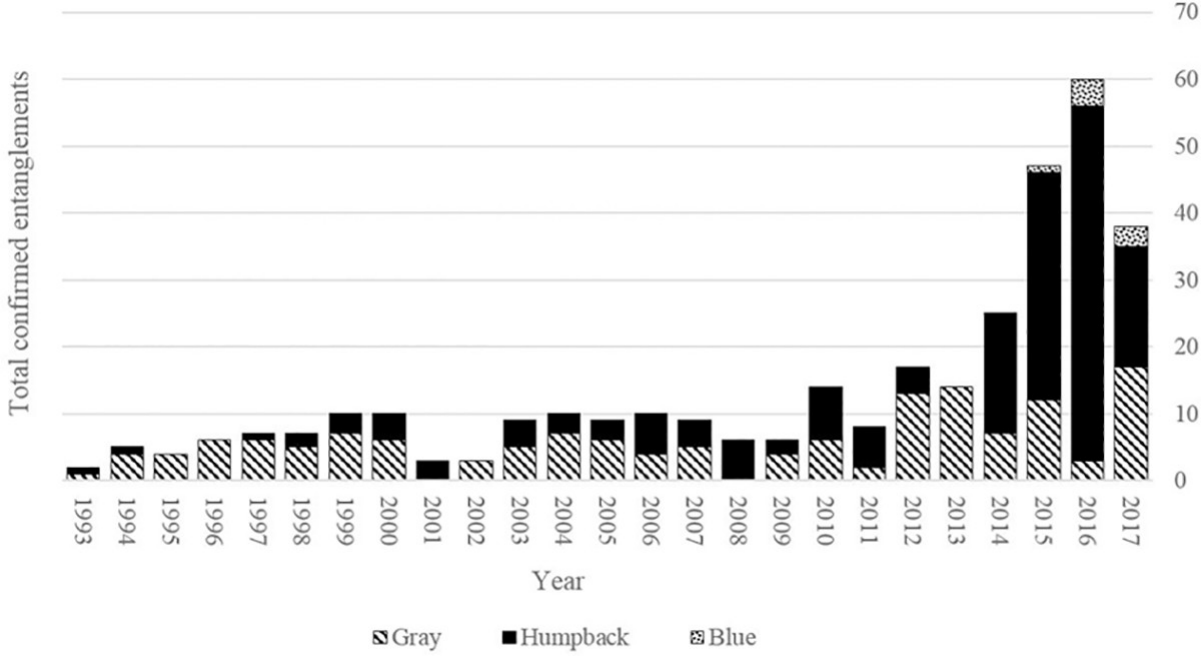
Total number of confirmed humpback (solid black), blue (dotted pattern) and gray whale (diagonal stripes) entanglements in fishing gear from 1993 to 2017 off the coast of California [32, Lauren Saez, pers. comm.].

However, indirect mortality remains a threat and a major anthropogenic threat facing baleen whales nowadays is entanglement in fishing gear [27]. Commercial fisheries are an important part of California’s economy. One of the most lucrative is the California Dungeness crab pot and line fishery [28], averaging about $75 million annually between the 2010/11 and 2017/18 seasons [29]. Recently, the rate of confirmed whale entanglements along the western coast of the United States increased dramatically from 8–10 per year in 1993–2010, to a record 60 in 2016 [30] (Fig 1). Though total entanglements dropped in 2018 (46 confirmed) and 2019 (26 confirmed), they are still higher than the pre-2014 average of about 10 entanglements per year [29]. The highest percentage of these entanglements were reported in the central California region [31]. Although mitigation strategies (i.e., education, gear alteration, and increased efforts by a disentanglement team) and concrete management actions have been in place since 2013 [29], entanglements continue to be a threat to local whale populations [31].

Predictive models that implement near real-time local oceanographic conditions can help mitigate baleen whale and fishery interactions [32, 33]. They can provide insights into baleen whale migration and distribution patterns that influence the risk of entanglements in central California [34, 35]. Generalized Linear Modeling (GLM), relating baleen species sightings data (counts) to climate, oceanography and other environmental variables, can be a useful tool with which to develop statistical predictive models. GLMs have been used in marine mammal research to predict and relate timing to environmental variability at a variety of timescales [32, 34, 35].

**Fig 2:**
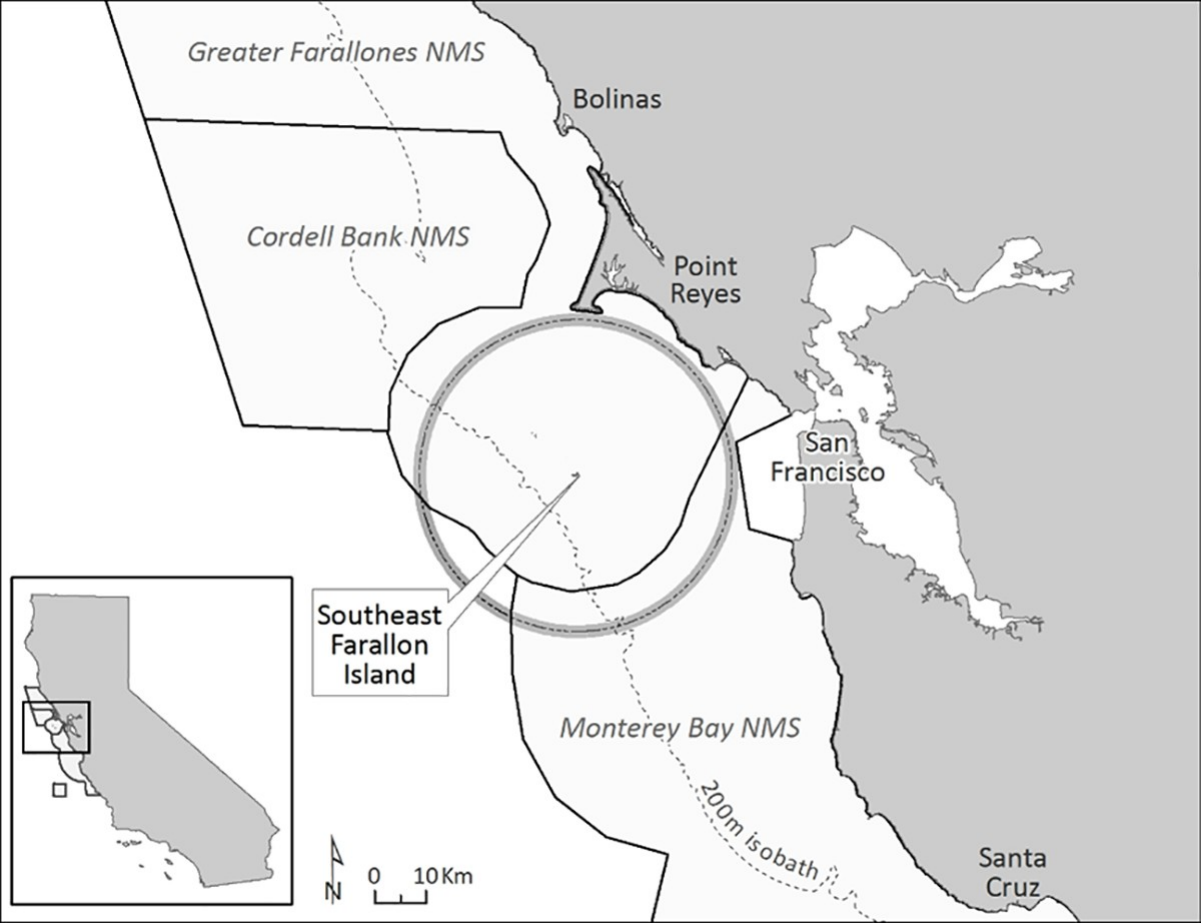
Map of the study area located off the coast of central California showing the location of Southeast Farallon Island and the 30km sight range. The Greater Farallones, Cordell Bank, and Monterey Bay National Marine Sanctuaries (NMS), which border the central California coast are outlined. The 200m isobath is depicted by the dashed line.

For this study, we used a 24-year time series of humpback, blue, and gray daily whale counts from Southeast Farallon Island (SEFI) to identify changes in local whale sightings, timing of local migration, and entanglement risk. A field station on SEFI, off the coast of San Francisco, operated by Point Blue Conservation Science in cooperation with the U.S. Fish and Wildlife Service, began systematically tracking sightings of blue, humpback, and gray whales in 1993 (Fig 2) [4]. Our goal was to explore possible reasons for the significant increase in entanglements in the study area. To determine this, we looked at how the number of overall sightings and the timing of whale arrival and departures have changed over time. For effective management, predicting the concentration of whales in a fishing area would be useful towards mitigating the overall risk of entanglements. We asked if there were any local, regional, or basin-scale environmental predictors of changes in arrival and departure time. We postulated that if the timing of migration could be accurately predicted, then these predictions can be applied to inform management actions to decrease entanglements in pot and trap fishing gear.

## Materials and methods

### Study area

SEFI (37’42”N, 123’01”W) is located 48 km off the coast of San Francisco in north central California. SEFI is the southernmost island of the seven rocky outcrops that make up the Farallon Islands National Wildlife Refuge, also within the Greater Farallones National Marine Sanctuary (Fig 2). Due to the nature of the data collected (see below), we recorded observations within 30km, the limit within which whales could be accurately identified to the species level (Fig 2).

### Species data collection

As part of an ongoing cetacean population study, systematic visual surveys were conducted daily from 1993 to the present by trained scientists from Point Blue Conservation Science [4]. All observations were recorded to the species level using 10X and 25X binoculars. Only positive observations at the species level were recorded and daily totals were conservatively estimated when large numbers were present [4]. We only included humpback, blue, and gray whale observations in this study because they accounted for about 99% of total baleen whale counts and were consistently observed throughout the time-series.

Additionally, standardized cetacean observation procedures were added in 2013 to implement new technology for data entry (Spotter Pro and Whale Alert Apps, 1515 N. Swinton Ave, Delray Beach, FL, 33444). Spotter Pro and Whale Alert are applications designed by Conserve.IO to report whale sightings along the west coast of the United States in real time to the National Oceanic and Atmospheric Administration (NOAA). This process is designed to inform NOAA, the U.S. Coast Guard, and commercial shipping vessels when large aggregations of whales are seen in the vicinity of shipping lanes (J. Jahncke, pers. comm.). Trained observers were employed to systematically count and record all observed cetaceans for an hour each day from the lighthouse on SEFI at an elevation of 90m [4], except during the gray whale winter migration when both morning and evening surveys were performed. For gray whales, the average of the morning and evening counts was used for the daily count. Observation days (subsequently referred to as “effort”) occurred when visibility was greater than 11.2 km, with no low-hanging fog, Beaufort wind scale was less than or equal to 4, and swells were less than 3 m.

Opportunistic, daily whale observations on SEFI were initiated in 1987. However, consistent systematic protocol only began in 1993; therefore, our analyses begin with that year. The database of daily whale counts from 1993 to 2012 only included values when animals were detected (counts ≥ 1), hence, it was unclear if the lack of data on a given day represented zero detections (count = 0) or no-effort due to weather conditions. Days with no effort represented false zeros [36]; to exclude these days, in the absence of daily-effort information prior to 2013, we used data from 2013 to 2016, collected via the Spotter Pro App on SEFI, to determine associations between effort and weather. Then we used the resulting statistical associations to identify days before 2013 that were likely no-effort days. Weather data were collected *in-situ* by the biologist on the island and included percent cloud cover, visibility (miles), barometric pressure (millibars), air temperature (Celsius), daily precipitation (inches), wind direction (in degrees), wind speed (knots), swell height (feet), and swell direction. First, we performed a Classification and Regression Tree (CART) analysis to split the weather data into effort and no-effort predictors. For example, days with high swells were classified as having a low chance of effort, while high visibility days were classified as having an increased chance of effort. CART results were then input into a predictive model, resulting in the likelihood of effort estimated from 0–1 for each day. We used Optimal Cutoff analysis to determine the value in which predicted effort could be optimally classified as a 0 (no-effort) or 1 (effort) [37]. Days that had zero recorded counts and a predicted effort of zero were removed from the dataset, leaving only days where effort was likely to have been recorded. Daily counts were then summed over seven day periods, and these weekly counts were used in the analyses (see below). The use of weekly counts reduced residual variance. Distance sampling [38] would be a valuable approach in this situation, but distance measurements were not recorded before 2013.

Two ecological anomalies were observed in the blue whale dataset. In all years except 2014, blue whales were absent during the winter months. More specifically, the number of blue whales observed in January-February was near zero (0.01 per week; less than 0.1% of all sightings). For that reason, we exclude winter months from all subsequent analyses of blue whale counts. However, we note that in 2014 sighting in January and February increase by several orders of magnitude compared to all other years, averaging 26.4 per week; much greater values than in the subsequent spring, summer, and fall (average 4.0 per week). Because of the near-total absence of blue whales in the winter of the other 23 years, we present analyses of the spring, summer, and fall for blue whales.

In 2006, blue whales abandoned the area and only two were recorded in the entire year (cf. average of 141 per year in all other years). Therefore, neither arrival nor departure could be assessed. This year was considered an ecological anomaly and was removed from both the sightings and timing models.

Average blue and humpback whale counts during the course of the year followed a unimodal distribution curve with a peak in the summer, so analysis was performed for all years starting on January 1^st^. Gray whale counts within the year followed a bimodal distribution with peaks in January and March, corresponding to southward and northward migration respectively. We analyzed these two peaks separately. To avoid splitting the first migration phase between calendar years, we used an adjusted year where day 1 is June 1^st^, and analyzed these data separately. We refer to the January peak as the gray south-bound breeding migration peak (gray-south), and the March peak as the gray north-bound feeding migration peak (gray-north).

### Environmental variable processing

#### Local variables

In situ daily SST and sea surface salinity (SSS) values were collected by scientists on SEFI. Front Intensity Index (FII) values were calculated at 5, 10, 15, and 20 km radii from SEFI by obtaining the maximum absolute value of the remotely sensed SST gradient within each radius. FII values were derived from the Operational Sea Surface Temperature and Ice Analysis (OSTIA) [39] (Table 1).

**Table 1:** Oceanographic and climate data used as environmental covariates.

Variable (Unit)	Mean ± SD	Min-Max values	Description	Data Source
***Local oceanography***				
SST (°C)	12.49 ± 1.44	8.77–18.24	Avg. sea surface temperature	https://scripps.ucsd.edu/programs/shorestations/shore-stations-data/data-farallon/
SSS (PSU)	33.40 ± 0.56	26.81–34.27	Avg. sea surface salinity	https://scripps.ucsd.edu/programs/shorestations/shore-stations-data/data-farallon/
FII (°C/km)	0.0331 ± 0.0144	0.0084–0.0990	Avg. front intensity index	http://ghrsst-pp.metoffice.com/pages/latest_analysis/ostia.html
***Regional***				
UI (m^3^/s/100m)	108.62 ± 104.16	-283–409.5	Avg. monthly Upwelling Index	http://www.pfeg.noaa.gov/products/PFEL/modeled/indices/upwelling/NA/upwell_menu_NA.html
STB (day anomaly from day 90)	85.05 ± 20.57	50–122	Avg. spring transition date	http://www.ndbc.noaa.gov/station_history.php?station = 46013
***Basin scale climate***				
SOI (standardized index)	-0.20 ± 1.85	-6.7–5.2	Avg. monthly Southern Oscillation Index values	http://www.cgd.ucar.edu/cas/catalog/climind/soi.html
PDO (standardized index)	0.133 ± 1.14	-2.33–2.79	Avg. monthly Pacific Decadal Oscillation values	http://jisao.washington.edu/pdo/PDO.latest
NPGO (standardized index)	0.245 ± 1.25	-2.99–2.96	Avg. monthly North Pacific Gyre Oscillation values	http://eros.eas.gatech.edu/npgo/

#### Regional variables

The upwelling index (UI) was downloaded from the Pacific Fisheries Environmental Laboratory website and averaged for the coastal region from Big Sur (36°N 122°W) to Point Arena (39°N 125°W), as SEFI lies between these two locations (Table 1). The spring transition anomaly (STB) was determined based on wind strength and direction data from the NOAA buoy 46013 in Bodega Bay, after estimating Ekman transport and the relative cumulative upwelling for each year (Table 1).

#### Basin-scale climate variables

SOI is a measure of the difference in pressure between Darwin and Tahiti used to identify El Niño and La Niña events [40], PDO is a measure of SST anomalies in the Pacific Ocean north of 20° [10], and NPGO is a measure of sea surface height and associated with fluctuations in SSS and nutrients in the Northern Pacific [7] (Table 1). The response of whales to shifts in climate patterns exhibits a delay because whale residency near SEFI was associated with prey availability [41]. PDO, for example, alters upwelling favorable wind strength which drives the concentration of nutrients and overall productivity [42]. Krill takes advantage of these productive areas [5]. This process takes time to make its way up the food chain. We calculated lags of 1, 2, and 3 months to account for such potential delays [43, 44].

### Sightings

Whales observed near SEFI migrate and forage in small groups. Therefore, the count data were skewed towards low daily numbers and a large number of days with zero counts. We used negative binomial regression to determine how whale sightings changed through time. All statistical analyses were carried out with Stata 16.1 (Stata Corp., 2019). Negative binomial regression modeling is recommended when count variables have a high variance, i.e., are over-dispersed [36, 44, 45]. We modeled weekly whale counts as a function of year to determine overall trends through time, incorporated month (as a quantitative variable) to account for seasonality within the year, and tested for any interactions between year and month. These covariates were tested for linear, quadratic, and cubic relationships with the count data. The log number of on-effort days per week (see *Species Data Collection* for more details) was used as an offset to control for differences in effort days among weeks.

### Timing

Average annual arrival, peak, and departure times were calculated for each species by identifying the day on which the 10^th^, 50^th^, and 90^th^ percentiles of annual sightings were recorded. Residency, which refers to the number of days that whales were near SEFI, was determined by subtracting the arrival day of each year from the departure day. For the gray-south dataset, weeks 1–18 (June-Sept.) were not analyzed. There were on average 10 to 15 gray whales seen per week during the summer near SEFI; however, the same whales were likely counted repeatedly (J. Jahncke, pers. comm.). This small resident population present in the summer is not applicable to the migrating portion of the population, which is observed in the late fall, winter, and spring.

Environmental variables were averaged annually and seasonally (Dec-Feb; Mar-May; Jun-Aug; Sep-Nov). We calculated annual and seasonal environmental values for gray whales based on the adjusted gray whale year (see above). Arrival, peak, and departure times were used in linear models as the dependent variable and the environmental variables (including linear, quadratic, and cubic terms) were tested as the independent variables. Significant covariates with the appropriate transformation (quadratic or cubic if either was significant) were then added to a preliminary linear regression multivariable model, and backwards stepwise elimination was used to sequentially drop non-significant variables until all variables remaining in the model were significant (P<0.05; [46]). The transformation of the highest order (cubic, quadratic, linear) was used if significant, in which case all lower order terms for that variable were retained. We then used the variance inflation factor (VIF) to verify that predictor variables were not collinear (VIF<10, [47]). The significant retained variables in the final multivariable models were used to estimate timing for each species. We used Akaike Information Criterion to confirm that a more parsimonious model was not preferred to our final models. To depict the relationship of each timing variable to the respective environmental variables in the final models of arrival or departure, we used the margins command in Stata, which provides predicted values and 95% CI, which holding all other variables in the statistical model at their mean value.

We created a set of forecast models, as an exercise to test how much power our model had in predicting the timing of arrival, peak, and departure variables. The forecast model included only significant environmental variables that occurred before the whale migration period in that particular year, reducing the number of variables and the performance of these models. In addition, we performed a year-removal validation by running the full model for each year, one year at a time, with that year’s observation removed. Predicted values for each year were compared to the actual observed values and model results were compared to the predictive ability of the original full model.

### Entanglements

Species-specific entanglement records were obtained from 1993 to 2016 and grouped by month. These data were collected and managed by National Marine Fisheries Service West Coast Region. We used a set of regression models to determine relationships between observed monthly entanglements and monthly whale count data and timing of migration.

We fit linear models to identify associations between monthly entanglements (dependent variable), monthly whale counts, and timing related variables (i.e., arrival and departure) by year for humpback whales, which were the only species to show a significant change in entanglements over time (see *Results*). The linear regression was chosen for this model because the distribution of residuals analyzing entanglements per year were consistent with a normal distribution, rather than following a negative binomial distribution, as was the case for weekly whale sightings (as described above).

First, we modeled monthly entanglements as a function of year to determine trends through time. Then, we created a combined sightings and timing model to see how arrival, departure, and the whale count per month influenced the number of entanglements in each month. Preferred transformations for each covariate were input into a preliminary multivariable linear regression model, and backwards stepwise elimination was used to reduce the model until all remaining variables were significant. VIF was again used to test collinearity between variables (i.e., VIF<10).

To compare the relative contribution of each predictor variable in accounting for variation in linear models, we compared the square of the t statistic, since the variance in the dependent variable due to a predictor in a linear model is proportional to the square of the t statistic [48].

## Results

### Changes in local sightings

**Fig 3:**
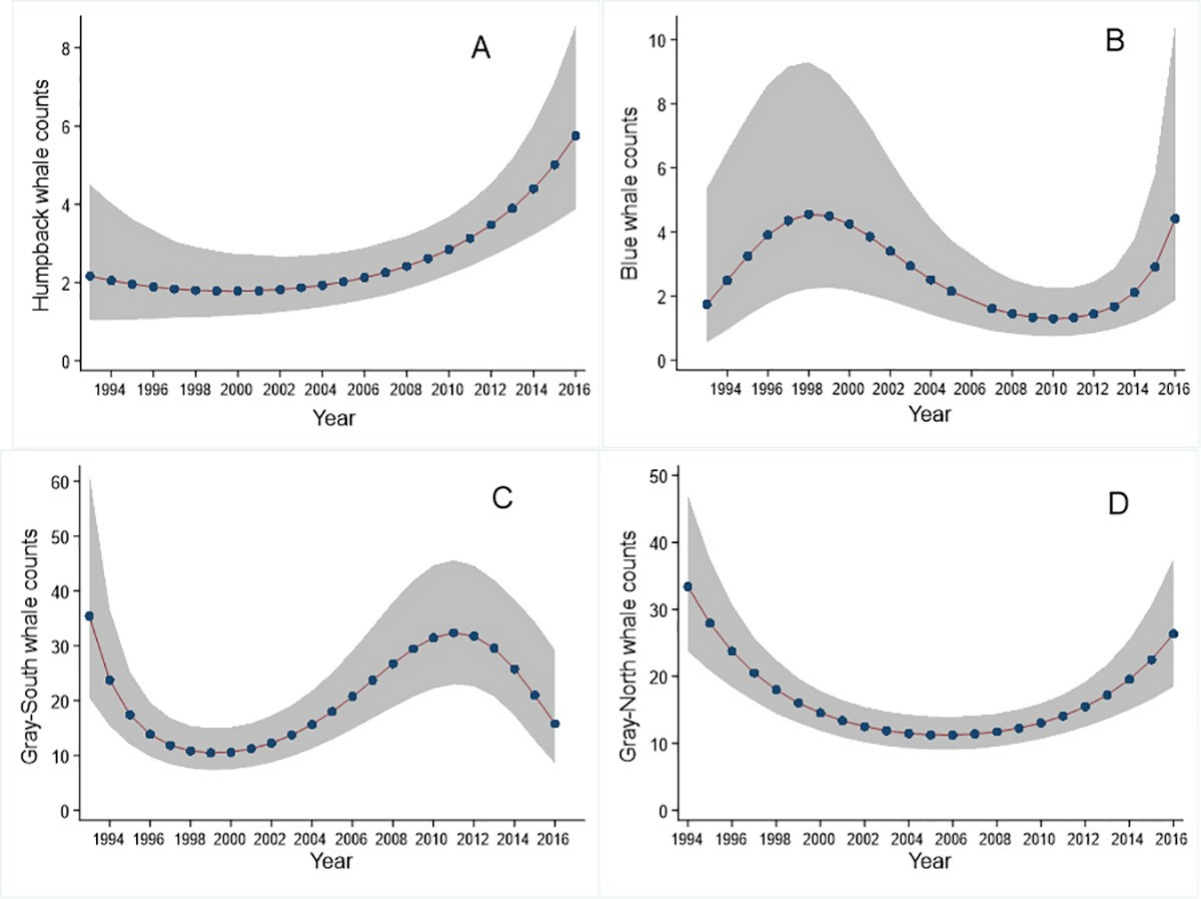
Interannual trends in average weekly predicted counts of (A) humpback, (B) blue, (C) south-bound gray, and (D) north-bound gray whales. The 95% confidence intervals are shown in gray shading.

Humpback, blue, and gray whale (both south and north) sightings by week showed non-linear trends with year (Negative Binomial Regression, humpback n = 1,217, blue n = 851, gray-south n = 320, gray-north n = 453). The modeled number of humpback whale sightings per week increased in a quadratic fashion, with little change from 1993 to about 2004 (about 2 per week), then accelerating, reaching six in 2016 (P<0.001 for the overall model, Fig 3A). Predicted blue whale sightings displayed a cubic trend, increasing between 1993 and 1998, from two to five, but then decreasing gradually until 2011 (P<0.001 for the overall model, Fig 3B). Between 2012 and 2016, sightings increased sharply from two to five. Gray-south and gray-north sightings per week showed similar sightings at the beginning of the time series with about 35 predicted sightings in 1993 (P<0.01 Fig 3C, P<0.001 Fig 3D). Both decreased to about 10 sightings but at different points, in the early 2000s for gray-south and in the late 2000s for gray-north. Gray-south sightings increased through the late 2000’s until about 2012 (30 sightings), but then dropped again to 15 sightings in 2016, thus displaying a cubic trend (Fig 3C). Gray-north sightings, instead, displayed a quadratic trend, starting to increase steadily from 2009, reaching about 30 sightings in 2016 (Fig 3D).

### Timing of local migration

**Fig 4:**
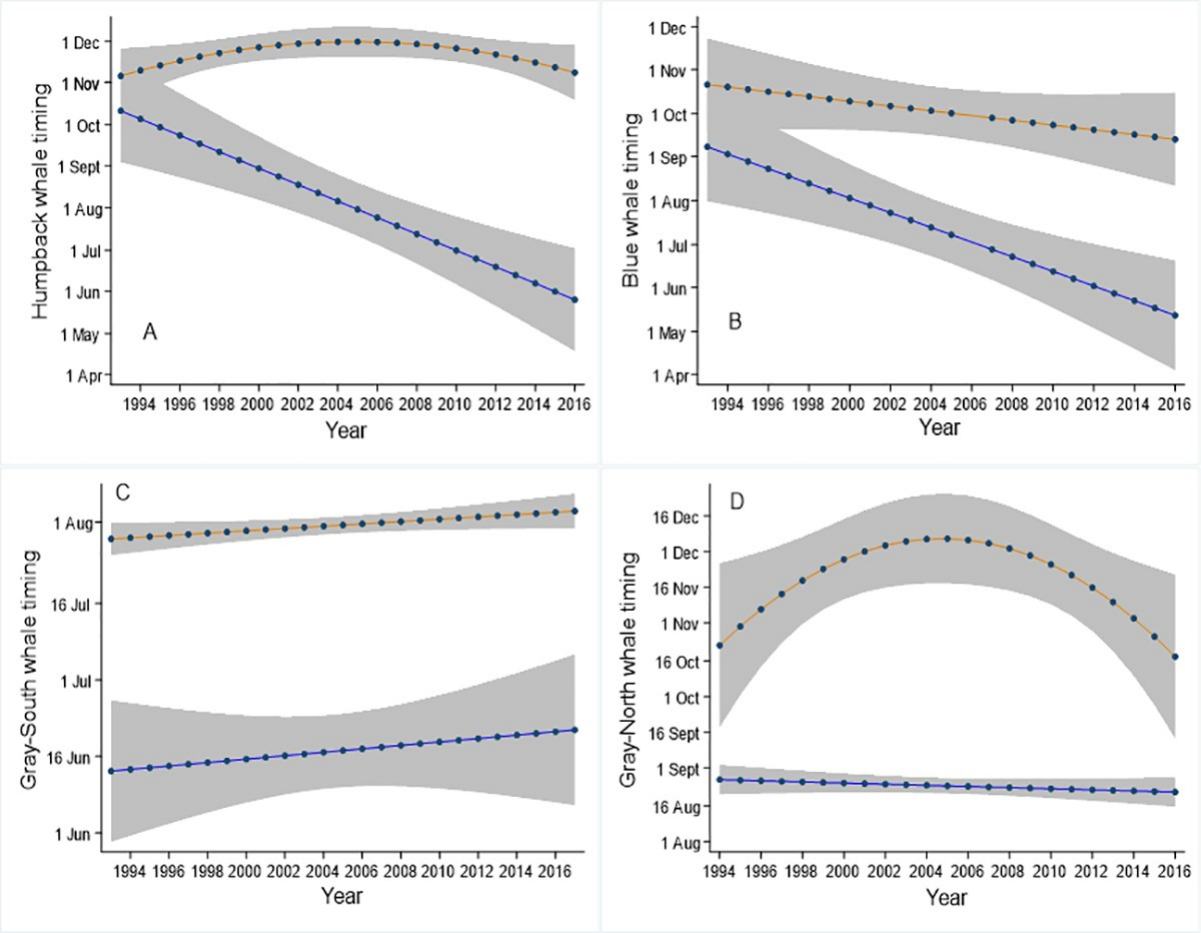
Interannual trends in the timing of day of arrival and departure. Shown are the arrival (blue) and departure (orange) trends for humpback (A), blue (B), gray-south (C), and gray-north (D). Blue whale departure, gray-north arrival, and gray-south arrival and departure, trends were significant (P>0.05); in this case we depict the linear trend. The other four trends shown were significant. The 95% confidence intervals are depicted in gray shading.

We found significant changes in timing of arrival for humpback and blue whales, as well as the timing of peak sightings and departure for humpbacks and gray-north. Here, we summarize patterns with regard to arrival and departure. Additional information about changes in peak times can be found in the (S1 Table in S1 File). All three species showed significant linear or quadratic trends for arrival and/or departure, but there were no significant cubic trends (Table 2, Fig 4). Humpback and blue whale timing of arrivals displayed linear trends over the time series and occurred, on average, 120 and 100 days earlier, respectively, comparing 2016 with 1993. Humpback whale timing of departure and gray whale departure dates during the feeding migration showed significant quadratic trends with year, representing a delay in the mid-2000’s, which was then reversed (Table 2). We found no significant association with year, for blue whale departure timing, gray-north arrival, or any of the gray-south migration metrics (Table 2, Fig 4).

**Table 2:** Changes in timing for humpback, blue, and gray whale arrival and departure times.

Species	Number of Years	Trend	P-Value
Humpback Arrival	24	L(-)	P<0.001
Humpback Departure	24	Q(-)	P<0.05
Blue Arrival	23	L(-)	P<0.01
Blue Departure	23	NA	Not Significant
Gray-south Arrival	24	NA	Not Significant
Gray-south Departure	24	NA	Not Significant
Gray-north Arrival	23	NA	Not Significant
Gray-north Departure	23	Q(-)	P<0.05

Trends are depicted as linear (L), quadratic (Q), or cubic (C). The coefficients for the highest order term in the model are depicted as positive (+) or negative (-).

The timing of migration for all species was influenced largely by basin-scale environmental variables, and less by local and regional variables, as described below (Summary of Linear Regression model statistics (Table 3): humpback n = 24, arrival and departure, P<0.001 for overall model; blue n = 23, arrival P<0.001, departure P>0.1, gray-south n = 24, arrival and departure P>0.1; gray-north n = 23, arrival P>0.3, departure P<0.001).

**Table 3:** Results from the multivariable timing model for humpback, blue, and gray whales.

Variable	Humpback arrival	Humpback departure	Blue arrival	Gray-north departure
SST			Q(-)*	
SST summer				L(-)***
SSS previous winter				Q(-)**
UI summer		L(-)*		
UI fall			C(+)*	
SOI previous spring	L(-)**			
SOI previous winter		C(-)**	C(-)***	
PDO	Q(-)***			
NPGO summer		Q(-)*		
Adjusted R^2^	0.6615	0.6808	0.7699	0.7011
P-value	0.0005	0.0001	0.0001	<<0.00001

Models of blue departure, gray-north arrival, and both gray-south metrics were not significant (P>0.05), and so are not shown.

Relationships are depicted as linear (L), quadratic (Q), or cubic (C). The coefficients for the multivariable model were depicted as positive (+) or negative (-). The most dominant variables (see text) are shown in gray shading. The level of significance is depicted by *** P≤0.001

** P≤0.01

* P≤0.05.

#### Local drivers

**Fig 5:**
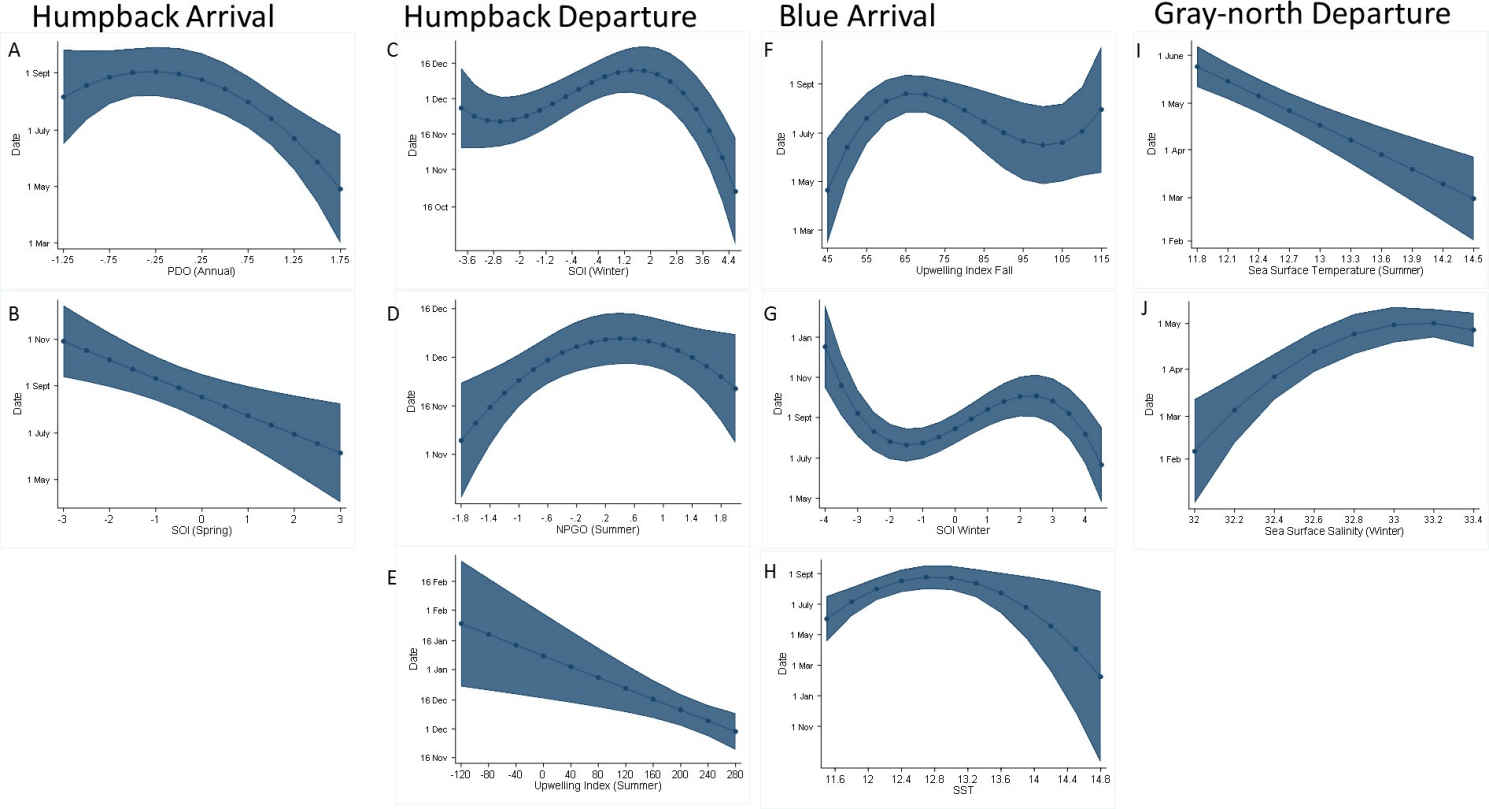
Visual depiction of the environmental multivariable timing models for humpback, blue, and gray whales. For each timing model shown in Table 3, the model predictions for each environmental variable is graphed while controlling for all the other significant variables in the model. Humpback arrival: SOI previous spring (A), NPGO-summer (B); Humpback departure: UI summer (C), SOI previous winter (D) NPGO summer (E); Blue arrival: annual SST (F), UI fall (G), SOI previous-winter (H); Gray-north departure SST summer (I), SSS previous winter (J). Shading indicates 95% CIs.

FII was not a significant driver in any of the models and SSS was only significant in the gray-north departure model (Table 3, Fig 5J). SST was a significant variable in blue arrival, indicating early arrival when the annual temperature was warmer (Fig 5F). Gray whales departing to feed left the area earlier when SST was warmer in the summer; this variable was the most important for gray-north departure (P < 0.001, Fig 5I).

#### Regional drivers

UI as a regional average was significant in humpback departure and blue whale arrival (Table 3, Fig 5C and 5G). However, in neither species-specific model was it the most significant driver. STB was not significant in any model (Table 3).

#### Basin-scale drivers

Among the basin-scale variables, SOI was the most commonly selected variable, achieving significance in all models except for gray whales on their feeding migration. High SOI values were associated with early arrival in both humpback and blue whales (Fig 5A and 5H). SOI in the previous winter was the most significant variable for blue whale arrival (P < 0.001) and humpback departure (P < 0.01). Annual PDO was the most significant variable explaining humpback arrival (P < 0.001), but NPGO also was significant in the humpback departure model (P < 0.05). Humpbacks arrived earlier during years of cool phase PDO (Fig 5A), and departed later when NPGO values were neutral to higher (Fig 5D).

For most environmental variables (local, regional, and basin-scale) seasonal averages were significant and were retained in the statistical models (Table 3). However, for SST and PDO, annual averages were significant and retained when analyzing blue arrival and humpback arrival, respectively (Table 3).

#### Forecast timing and validation

Timing of migration models for humpback, blue and gray whales were used to forecast arrival and departure times. Considering only the four timing models that were significant (see above), all four forecast models were significant as well (humpback arrival and departure, P < 0.001; blue arrival P < 0.05; gray-north departure, P < 0.05). The forecast models explained 34–60% of the variance in the original, full model (Table 4).

**Table 4:** Coefficient of determination and model significance comparison between the full, forecast, and year-removal validation models.

	Full Model		Forecast Model		Year Removal Validation		Ratio: Forecast to Full Model
	R^2^	P-value	R^2^	P-value	R^2^	P-value	
Humpback Arrival (n = 24)	0.662	P<0.001	0.458	P<0.001	0.471	P<0.001	0.599
Humpback Departure (n = 24)	0.681	P<0.001	0.681 No variables removed	P<0.001	0.588	P<0.001	1.0
Blue Arrival (n = 23)	0.770	P<0.001	0.263	P<0.050	0.574	P<0.001	0.342
Gray-North Departure	0.701	P<0.001	0.290	P<0.050	0.579	P<0.001	0.414
(n = 23)							

Only results from statistically significant timing models are shown here.

Predictive models were validated by a year removal test, predicting timing for each year based on a model fit without that year’s data. The year-removal models showed good ability to predict that year’s value (R^2^ varied from 0.471 to 0.588; P < 0.001 for all four models; Table 4). Proportionately, the R^2^ of the year-removal model was between 62 and 85% of the R^2^ of the full-data model.

### Entanglement risk

Humpback whales were the only species that demonstrated a significant change in the total number of entanglements over the time series. There were substantially more entanglements of this species than either of the other two. Therefore, humpback whale entanglement models are the only species reported here.

We found that both the total number of whales and the timing of arrival significantly influenced the number of entanglements (Linear Regression; n = 75, P<0.05 for each variable; P < 0.001 for the multivariable model, Table 5). The total number of whales had a greater influence on total number of entanglements than the timing of arrival, as indicated by the square of the t statistic for humpback sightings (t^2^ = 10.24) compared to that of humpback arrival (t^2^ = 5.02). Thus the number of whales observed per month account for 104% more of the variance in entanglements than do humpback arrival dates.

**Table 5:** Results from the multivariable model for confirmed entangled humpback whales.

***Variable***	
Humpback Counts	L(+)*
Humpback Arrival Day	L(-)*
***Model Statistics***	
Adjusted R^2^	0.1604
P-value	0.0007

Relationships are depicted as linear (L), quadratic (Q), or cubic (C). The coefficients for the multivariable model are depicted as positive (+) or negative (-). The level of significance is depicted by ***P≤0.001, ** P≤0.01

*P≤0.05.

## Discussion

We found that humpback whale model-predicted sightings increased from 1993 to 2016, blue whale sightings fluctuated between two to five average sightings per week, and both species showed a significant change in arrival time to central California (Figs 3 and 4). Gray whales displayed significant trends in sightings for both north-bound and south-bound migration (Fig 3). Gray whales did not have a significant change or timing of arrival, although there was a significant change in the timing of the departure of northbound gray whales (Fig 4). In the northwestern Pacific Ocean, humpback and gray whales have continued to recover from whaling in the last few decades [31, 49]. In addition, blue whale population estimates have been reported to have increased significantly from 2014 [31, 50]. Although our findings displayed variability, predicted blue whale counts were similar at the beginning and end of the time series (Fig 3B) though they do indicate an increase in the most recent years. Our data were limited in spatial extent and should be used to describe local population trends only. Interannual variation in both the number of whales and timing of migration has been reported in baleen whales previous to this study [3, 22, 51], but no trend has previously been documented in this area.

Our findings are unique because they reveal a significant trend towards earlier arrival over three decades in this important feeding area (Fig 4). We found that humpback whales arrived on average 120 days earlier, while blue whales arrived in central California 100 days earlier in 2016 than they did in 1993. Similar changes in the timing of migration have been observed in southern California [51]. Short term, interannual changes in whale species composition and arrival have been previously documented in response to changes in the environment [3]. Baleen whales have been reported to respond to changes in prey availability [3, 18, 52]. These lower trophic levels are highly susceptible to changes in the environment. Thus, this study has connected changes in the physical environment to altered migration patterns.

Throughout our study period, there has been variation in local, regional, and basin-scale environmental conditions. Each species had a unique environmental driver that contributed the majority of the variation in the timing of migration (Table 3). The most common environmental drivers were local SST, regional Upwelling Index (UI), and basin scale climate indexes: Southern Oscillation Index (SOI), Pacific Decadal Oscillation (PDO), and North Pacific Gyre Oscillation (NPGO). The main environmental drivers for each species occurred on different spatial scales. Humpback arrival and departure were mostly driven by basin-scale variables, gray-north departure by local oceanography, and blue arrival by a combination of variables from local to basin-scale (Table 3). The importance of these variables to whale behavior mirrors findings in previous studies of the CCS [3, 20, 22]. Climate patterns that were associated with increased SST and generally unproductive conditions (Fig 5), showed a strong correlation with changes in timing of all three species.

The most dominant predictor of humpback arrival was annual PDO, with SOI also contributing to the model. Early arrival occurred during warm, non-productive years, indicated by PDO values (Fig 5A) following a productive year as indicated by SOI in the previous year (Fig 5B, Table 3). The humpback departure model was significant and varied by less than 30 days over the study period (Fig 4A). Variation was most strongly driven by summer variables (NPGO and upwelling), with previous year conditions (SOI-winter) contributing as well. Both positive NPGO and cool phase PDO are often strong indicators of overall ocean productivity [6, 7]. In short, the most significant drivers of humpback timing were climate indices that reflected low productivity. Observed differences in humpback sightings from SEFI may be explained by prey switching, which led to changes in feeding locations, such as onshore or offshore habitat use, during the study period [53]. The associations we found in the species-specific models demonstrate how climate patterns affect this system, prey availability, and humpback whale residency.

We found the most significant driver of blue whale arrival time was lagged by nearly a year but local variables (fall upwelling and annual SST) also contributed to the model (Table 3). Early blue whale arrival occurred during warm, non-productive years, as indicated by high annual SST (Fig 5H) and low seasonal upwelling (Fig 5F) following a winter with low productivity (Fig 5G). This significant lag in the driver of early arrival is likely due to the importance of krill biomass in an area [51] and may also be attributed to blue whale memory [54]. Unproductive environmental conditions, such as characterized by strong El Niño years (negative SOI) or increased SST, reduce productivity and contribute to changes in blue whale migration patterns [50, 55].

Gray-north departure was earlier when summer SST was warm, following a winter with increased freshwater input possibly due to increased rain or weak upwelling (Fig 5I and 5J).

Baleen whale residency in an area is considered to be strongly influenced by prey availability [3, 24]. Our results support this assumption. The drivers of whale departure day were local variables such as SST and Upwelling Index (UI), which are associated with prey biomass [56]. The warmest period of SST in our study was from 2014–2016 due to a combination of a strong marine heat wave (also referred to as “the blob”), an anomalous warm patch of water that circulated the northeastern Pacific, and a very strong El Niño event in 2015 [57, 58]. Large whales are highly mobile, and able to travel to optimal conditions where food is most abundant [20]. While the CCS was less productive than normal during this time period, productivity in central California was greater than in southern California [57]. Conditions in southern California, such as low UI and warm SST, likely reduced prey biomass. Possibly, some whales continued to more favorable feeding grounds in central California rather than stay in unproductive feeding grounds in southern California [57]. As both humpback and blue whale populations are recovering [49], increased sightings near SEFI in recent years may indicate higher concentrations of whales in the central California feeding ground (Fig 3).

### Entanglements

**Fig 6:**
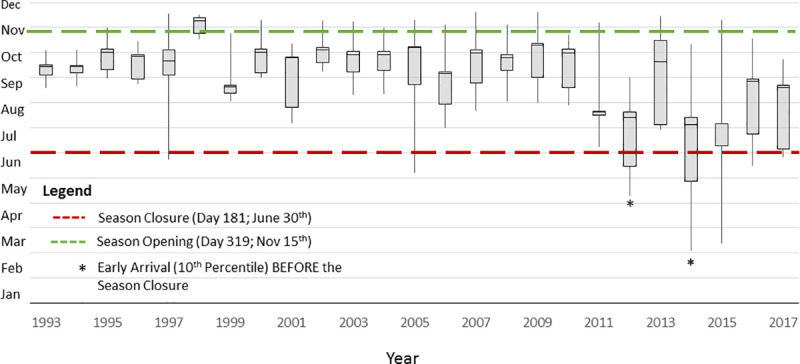
Residency time of humpback whales within 30 km of the Southeast Farallon Islands. The bottom of the box plot corresponds to the arrival time (date of 10^th^ percentile for the year’s sightings). The top of the box plot corresponds to the departure time (date of 90^th^ percentile of the year’s sightings). The whiskers are the earliest arrival date (bottom) and the last departure date (top). The red line corresponds to the typical closure of the previous year fishing season on June 30^th^ (Day 181) and the green line is the typical opening of the new fishing season on November 15^th^ (Day 319). Years marked with a * correspond to years where there was early arrival.

The environmental variables that drive earlier arrival must be considered with respect to effective management, as whales will be exposed to increasing anthropogenic risk and competition with humans over time [54, 59]. SEFI is located near the heavily urbanized San Francisco Bay. Therefore, it is critical to understand patterns of whale sightings and timing within the context of associated anthropogenic threats. Pot and trap fishing gear was the most common type of gear identified in all entanglements, and the California Dungeness crab fishery was responsible for the majority of those [30, 31]. This fishery historically was open from mid-November to the end of June [28] (Fig 6). In our time series, we found that, on average, humpbacks arrived in early August and departed in mid-November. These arrival and departure times occurred when the crab fishery was closed. Under typical past conditions and migration timing, there was thus little overlap between whales and the pots, which resulted in a relatively low number of humpback entanglements (Fig 6).

Monthly humpback entanglements increased as counts of humpbacks increased. In addition, monthly entanglements increased in years with earlier arrival. While our models showed entanglements in humpbacks associated more with increased sightings, we also saw a strong connection between timing of arrival and entanglements. When whales arrive to the area early, the number of sightings also increased. Our results showed that increased sightings as a result of early arrival increased the concentration of whales in the area which appears to lead to more entanglements. Since 1993, the first humpbacks were observed before the closure of the fishery during the El Niño of 1997/98, the ocean anomaly of 2005, and since 2012. The exception to that pattern was observed in 2013, when humpback whales arrived after the closure of the fishery. There were no recorded humpback entanglements in that year, providing support of a causal relationship.

It is important to note that in 1997 and 2005 there were no unusual increases in entanglement rates. However, humpbacks were first observed only 10 days before the fishery closure in 1997 and 35 days before the 2005 closure (Fig 6). More importantly, our arrival metric (10^th^ percentile of sighting) in those two years was not unusually early (Fig 6). Thus, this greatly reduced the chances of the whales interacting with the pots while they were still in the water. In 2014 and 2015, humpbacks were exposed to the lines for about three times as long, so duration of exposure was likely an important factor in the total entanglement number. While gray whale entanglements have been relatively consistent through time, humpback entanglements dramatically increased from an average of less than 10 per year before 2014 to five times that in 2016 (Fig 1). Unusually high humpback entanglements were observed annually since 2014, which corresponds to the earliest arrival times and higher sightings.

SST at SEFI gradually increased from 11.9°C in July of 1993 to 12.2°C in July of 2016. These conditions are typically associated with prey switching behavior in humpbacks [24] from krill, which aggregate on the shelf break [53], to forage fish, which migrate north during periods of warmer SST [60]. During the 2015/16 season, a domoic acid outbreak in the Dungeness crab fishery delayed the opening of the season in some areas along the coast of Northern California, so pots were aggregated in a smaller area, closer to shore [56]. Not only were humpbacks observed feeding on forage fish closer together in areas with a high concentration of crab pots [53], but more humpbacks were in the region due to early arrival (Fig 6). All of these factors likely contributed to the dramatic spike in humpback entanglements in 2015–2016.

Only five blue whale entanglements were confirmed in central California through 2016, so statistical analysis was not possible due to the low sample size. All recorded blue whale entanglements have been since 2015. Blue whales feed primarily on krill, which typically aggregate along the shelf-break [56]. Even as blue whales arrived earlier to the area (Fig 4), they would have spent the majority of their time away from the near-shore cluster of crab-pots [53]. However, blue whales still would have had a longer period of overlap with the fishery due to the population arriving early. As these data continue to be collected, future blue whale entanglements will inform us if these few entanglements were isolated incidences, or represent an emerging trend.

We found that gray whales were most commonly seen in the area from December to April, while the crab fishery was open. Gray whales have had consistent interactions with this fishery for the entirety of the time series during these months. This may explain why we found roughly the same number of annual gray whale entanglements through the time series (Fig 1). However, other studies have found that their migrations are changing, similar to the humpback and blue whales we observed [22, 43, 61], so it is important to continue to monitor the entanglement risk that this population faces.

To mitigate the local risk of entanglements, the California Department of Fish and Wildlife closed the Dungeness crab fishery in April in 2019 and 2020. This new earlier closure was intended to limit temporal overlap with whales and result in less entanglements overall. In 2019, there were 26 confirmed entanglements on the west coast [29]. Though less than the 2015 peak, these data are higher than the pre-2013 average.

Predictive models, such as these, can be used to predict arrival and departure dates in advance allowing managers to adjust the length of the fishing season to reduce the temporal overlap with whales. This may decrease entanglement risk. Forecast models were shown to have high predictive value, especially for humpback arrival and departure (Table 4). Based on these results, we can effectively predict humpback arrival and departure in advance.

While we found significant trends in arrival dates (correlated with higher risk), it is difficult to determine whether those will continue. Earlier arrival of humpback and blue whales appears to be a response to a combination of warming oceans and associated changes in prey availability. The waters near the Farallones have warmed; SEFI SST gradually increased through our time series with the highest average monthly temperature recorded in August of 2014 at 17.04°C. If this ocean warming trend continues as a result of climate change, the unprecedented entanglement rates of whales are likely to continue with negative consequences to whale populations in central California. Long-term, real time monitoring of whale behavior and oceanographic conditions in central California and optimally, across the entirety of the species ranges, is critical for the management and protection of these species.

## Supporting information

S1 File (DOCX)

S1 Fig Visual depiction of the environmental multivariable timing models for humpback, blue, and gray whales. For each timing model shown in S2 Table in S1 File, the model predictions for each environmental variable is graphed while controlling for all the other significant variables in the model. Humpback Peak: PDO fall (A), SOI spring (B) UI fall (C), annual SST (D) Gray-north peak: annual NPGO previous-year (E) SOI winter (F) Shading indicates 95% CIs.
(TIF)
