# Supplementary material for: Modeling changes in baleen whale seasonal abundance, timing of migration, and environmental variables to explain the sudden rise in entanglements in California
Source: PLoS One. 2021 Apr 15;16(4):e0248557. doi: 10.1371/journal.pone.0248557 (PMC8049321; doi:10.1371/journal.pone.0248557)
Supplement: S1 File — (DOCX) [file pone.0248557.s001.docx]

Peak results are displayed in tables similar to the tables found within the manuscript.

**S1 Table. Changes in timing for humpback and gray-north peak times.** Trends are depicted as linear (L), quadratic (Q), or cubic (C). The coefficients for the model were depicted as positive (+) or negative (-).

| **Species** | **Number of Years** | **Trend** | **P-Value** |
| --- | --- | --- | --- |
| Humpback Peak | 24 | Q(-) | P<0.05 |
| Blue Peak | 23 | NA | Not Significant |
| Gray-south Peak | 24 | NA | Not Significant |
| Gray-north Peak | 23 | Q(-) | P<0.01 |

**S2 Table. Results from the multivariable peak timing model for humpback and gray-north whales.** Relationships are depicted as linear (L), quadratic (Q), or cubic (C). The coefficients for the multivariable model were depicted as positive (+) or negative (-). The most dominant variables (see text) are shown in gray shading. The level of significance is depicted by *** P≤0.001, ** P≤0.01, * P≤0.05.

| **Variable** | **Humpback peak** | **Gray-north peak** |
| --- | --- | --- |
| ***Local oceanography*** | | |
| SST | L(-)* |  |
| ***Regional upwelling*** |  |  |
| UI fall | C(+)** |  |
| ***Basin-scale climate*** | | |
| SOI spring | C(-)** |  |
| SOI winter |  | L(+)* |
| PDO fall | Q(-)** |  |
| NPGO previous year |  | L(+)** |
| ***Model Statistics*** | | |
| Adjusted R^2^ | 0.7607 | 0.4327 |
| P-value | 0.0002 | 0.0009 |

**S3 Table. Coefficient of determination and model significance comparison between the full, forecast, and year-removal validation models. Only results from statistically significant timing models are shown here.**

|  | Full Model | | Forecast Model | | Year Removal Validation | |
| --- | --- | --- | --- | --- | --- | --- |
|  | R^2^ | P-value | R^2^ | P-value | R^2^ | P-value |
| Humpback Peak | 0.761 | P<0.001 | 0.233 | P<0.001 | 0.581 | P<0.001 |
| Gray North Peak | 0.453 | P<0.001 | 0.268 | P<0.050 | 0.320 | P<0.050 |
