# Supplementary figures and images for: Modeling changes in baleen whale seasonal abundance, timing of migration, and environmental variables to explain the sudden rise in entanglements in California
Source: PLoS One. 2021 Apr 15;16(4):e0248557. doi: 10.1371/journal.pone.0248557 (PMC8049321; doi:10.1371/journal.pone.0248557)

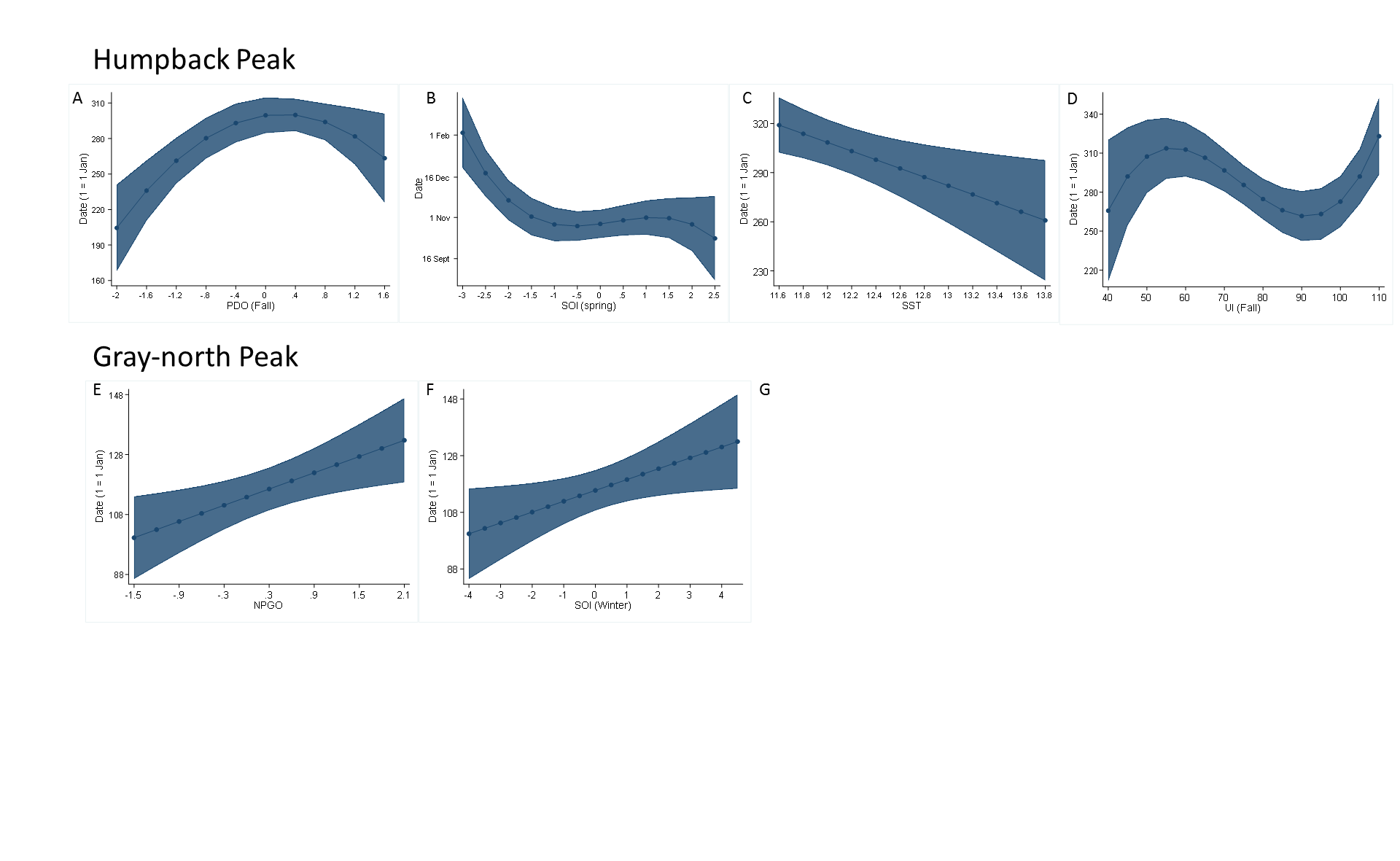

Supplement: S1 Fig — For each timing model shown in S2 Table in S1 File, the model predictions for each environmental variable is graphed while controlling for all the other significant variables in the model. Humpback Peak: PDO fall (A), SOI spring (B) UI fall (C), annual SST (D) Gray-north peak: annual NPGO previous-year (E) SOI winter (F) Shading indicates 95% CIs. (TIF) [file pone.0248557.s002.tif]
